# “Looking inward,” creativity, and well-being: an SEM analysis of self-reflective rumination and creative self-efficacy as pathways linking self-beliefs in creativity and well-being in college students

**DOI:** 10.3389/fpsyg.2026.1776026

**Published:** 2026-05-28

**Authors:** Dongdong Liu, Chenggang Wu, Jing Dang

**Affiliations:** 1 College of Teacher Education, Suqian University, Suqian, China; 2 Key Laboratory of Multilingual Education with AI, School of Education, Shanghai International Studies University, Shanghai, China; 3 Institute of Language Sciences, Shanghai International Studies University, Shanghai, China; 4 College of Education, Inner Mongolia Normal University, Hohhot, China

**Keywords:** creativity, creativity self-efficacy, self-belief in creativity and well-being, self-reflective rumination, well-being

## Abstract

Building on prior evidence linking self-reflective rumination to creativity, and given the well-established association between creativity and well-being, the present cross-sectional study examined associations between self-reflective rumination and self-beliefs in creativity and well-being (SBCW), with specific attention to the potential indirect role of creative self-efficacy. A large sample of Chinese college students (*N* = 1993) completed validated questionnaires assessing self-reflective rumination, SBCW (encompassing short-term and long-term dimensions), and creative self-efficacy. Structural equation modeling indicated that self-reflective rumination was significantly and positively associated with short-term SBCW. Furthermore, creative self-efficacy demonstrated a significant indirect association between self-reflective rumination and SBCW across both temporal dimensions, suggesting that constructive self-reflection correlates with stronger beliefs in the positive association between creativity and well-being. These findings highlight robust correlational patterns involving self-reflective rumination, creativity-related beliefs, and psychological well-being. The results also suggest that creative self-efficacy may be concurrently linked to reflective cognitive processes alongside creative behavior. The present study further supports the Creative-Being Model, which conceptualizes self-reflection, creativity, and well-being as interconnected components of human flourishing.

## 1 Introduction

Humans frequently engage in self-reflection, contemplating their experiences, inner feelings, and thoughts. However, for some individuals, this reflective process becomes repetitive, rigid, and narrowly focused on negative emotions and distress, along with their causes and consequences. This maladaptive cognitive style is known as rumination (Nolen-Hoeksema et al., 1999; Townshend and Hajhashemi, 2025). A substantial body of evidence has established a strong positive association between rumination and prolonged negative mood, as well as an increased risk of depression (Nolen-Hoeksema et al., 2008; Nolen-Hoeksema and Morrow, 1991; Tamura et al., 2025). Nolen-Hoeksema (1987) further proposed rumination as a key explanatory factor for the higher prevalence of unipolar depression in women compared to men, suggesting that women are more likely to respond to distress with ruminative thinking, whereas men tend to engage in more active coping strategies. Subsequent research has shown that rumination not only amplifies negative thinking but also impairs problem-solving abilities and reduces the likelihood of seeking or receiving social support (Nolen-Hoeksema, 1987). However, Nolen-Hoeksema (1987) also acknowledged that not all forms of self-focused thought are maladaptive. This opens space for distinguishing rumination from adaptive self-reflection (Shigemoto, 2022). This distinction is notably advanced in the work of Trapnell and Campbell (1999), who differentiated between ruminative self-focus (maladaptive) and intellectual self-reflection (adaptive). They found that intellectual self-reflection is positively associated with openness to experience, a core personality trait linked to curiosity, imagination, and aesthetic sensitivity (Trapnell and Campbell, 1999). Importantly, this adaptive form of self-reflective thinking has been linked to enhanced creativity (Verhaeghen et al., 2014).

### 1.1 Self-reflective rumination and creativity

Drawing from the canonical definition of creativity, Runco and Jaeger (2012) posited that creative expression encompasses two essential dimensions: originality and effectiveness. Originality entails the manifestation of ideas or artifacts that are novel, unprecedented, idiosyncratic, or distinctively unconventional. To engender such originality, individuals may need to engage in self-reflective rumination, an inwardly directed, metacognitive process that facilitates the generation of innovative conceptions by challenging presuppositions and reconceptualizing conventional paradigms. As argued by Verhaeghen et al. (2005), rumination may be linked to creativity as a distinct cognitive style, a tendency particularly evident among writers, painters, filmmakers, and other artists. These individuals often use their art to express, process, and shape their emotions and inner experiences, a process rooted in self-reflection. Rather than merely dwelling on distress, they transform introspective thought into creative output, suggesting that ruminative self-focus, when channeled adaptively, can serve as a catalyst for artistic and intellectual innovation (Verhaeghen et al., 2005). Based on this speculation, Verhaeghen et al. (2005) explored how self-reflective rumination was related to creativity and observed that self-reflective rumination was positively associated with various dimensions of creativity, including creative interests, fluency, originality, and elaboration. The positive association between self-reflective rumination and creativity has been replicated by many later studies (Cohen and Ferrari, 2010; Escobar and Perez, 2023; Forgeard, 2013; Verhaeghen et al., 2014). For example, Cohen and Ferrari (2010) found that creativity was predicted by reflective rumination and this prediction was modulated by indecision. Specifically, reflective rumination predicted creativity for individuals with high indecision, and there was no relationship between reflective rumination and creativity for individuals with low indecision. Moreover, given that rumination encompasses both adaptive and maladaptive forms, Verhaeghen et al. (2014) showed that differentiating these subtypes elucidates its associations with creativity and with dysphoria or depression, outcomes that earlier studies have consistently linked to rumination (Mor and Winquist, 2002; Tamura et al., 2025). Using the differential rumination measure distinguishing self-reflective pondering from brooding (Treynor et al., 2003), Verhaeghen et al. (2014) examined how these two forms relate to creative performance and depressive symptoms. Results revealed that self-reflective pondering (a purposeful and problem-focused form of reflection) was positively associated with creative behaviors but showed no significant link to dysphoria. In contrast, brooding (a passive and repetitive comparison of one’s current state with unmet goals) was strongly and positively associated with dysphoria, but not with creativity. These findings not only highlight a critical distinction between the two dimensions of rumination but also support the idea that self-reflective rumination can have adaptive, even beneficial, effects on cognitive processes such as creativity.

Vahle-Hinz et al. (2017) demonstrated that rumination can also serve as a facilitator of innovation in professional settings. The study distinguished between two work-related forms of rumination: one tied to negative emotional states and the other focused on reflective problem-solving strategies. Results showed that problem-related pondering, a constructive, goal-oriented form of reflection, at Time 1 (spring 2013) positively predicted creativity at Time 2 (spring 2014), suggesting that self-reflective rumination enhances creative outcomes in the workplace. This finding extends prior evidence to a longitudinal, real-world context, reinforcing the adaptive potential of reflective thinking (Vahle-Hinz et al., 2017). More recently, Ahmadi et al. (2025) expanded this line of research by examining how different types of rumination relate to emotional creativity and personal growth, a key dimension of well-being. Their findings revealed that both deliberate (intentional, self-reflective) and intrusive (involuntary, repetitive) rumination predicted emotional creativity, but in opposite directions: deliberate rumination was positively associated with emotional creativity, whereas intrusive rumination showed a negative association. Moreover, deliberate rumination significantly predicted cognitive creativity, while intrusive rumination did not (Ahmadi et al., 2025). These results underscore the critical distinction between adaptive and maladaptive forms of rumination, aligning with prior research (Cohen and Ferrari, 2010; Escobar and Perez, 2023; Forgeard, 2013; Verhaeghen et al., 2014). Notably, neither form of rumination directly predicted posttraumatic growth. However, deliberate rumination indirectly predicted growth through the mediating role of cognitive creativity, suggesting that reflective thinking fosters well-being not directly, but via its contribution to creative cognition. This pathway highlights creativity as a psychological resource linking rumination to positive development. The well-established connection between creativity and well-being will be further discussed in the following section.

### 1.2 Creativity, well-being, and self-beliefs in creativity and well-being (SBCW)

As Kaufman (2018) observes, prior research has predominantly centered on strategies to enhance creativity, yet comparable attention deserves to be directed toward investigating the benefits that creativity confers. It is well established that positive affect fosters creativity (Baas et al., 2008), a relationship thought to operate through mechanisms such as increased cognitive flexibility (Ashby et al., 1999) and enriched associative connections within knowledge structures (Lyubomirsky et al., 2005). In recent years, a growing body of research has confirmed that creativity, in turn, contributes positively to well-being, with findings spanning correlational, meta-analytic, and experimental studies.

For instance, Wright and Walton (2003) found a positive association between psychological well-being and creativity, even after accounting for the influence of positive mood, suggesting the relationship is not merely a byproduct of transient positive affect (Wright and Walton, 2003). Jean-Berluche (2024) further synthesized this literature in a review, highlighting a plausible link between creative expression and mental health: creative engagement, they argue, enhances emotional regulation, cognitive flexibility, and social connectedness, which collectively support well-being (Jean-Berluche, 2024). Complementing these findings, a meta-analysis by Acar et al. (2021) corroborated a robust positive relationship between creativity and well-being, noting that this association is stronger when creativity is measured via real-world creative activities compared to performance on divergent thinking tasks (Acar et al., 2021). Extending this line of inquiry, Li and Wu (2025) recently examined the potential mediating role of meaning in life: creativity is positively associated with well-being, and meaning in life partially accounts for this association, suggesting that creative pursuits correlate with a stronger sense of purpose, which is concurrently linked to greater well-being (Li and Wu, 2025). Beyond cross-sectional surveys, Smith et al. (2022) employed a daily diary methodology to examine dynamic associations among creativity, affect, and well-being in creative individuals. Their findings indicated that on days when creative adults reported higher levels of positive affect, their creativity and well-being scores tended to be correspondingly higher; conversely, higher negative affect was associated with lower creativity scores (Smith et al., 2022).

To further solidify the causal claim that creativity enhances well-being, robust evidence has emerged from experimental studies, moving beyond correlational designs to establish directional effects. For instance, Tan et al. (2021) first validated a positive association between creativity and well-being in two samples (undergraduates and working adults), even after controlling for demographic variables and stress. Building on this correlational foundation, they employed an experimental paradigm to test how creativity priming influences subjective well-being. Across two independent experiments, participants in the creativity-primed condition (e.g., engaging in tasks designed to activate creative thinking) consistently reported significantly higher subjective well-being compared to those in the control group. These findings directly support a facilitative, causal effect of creativity on well-being (Tan et al., 2021). Extending this line of inquiry, Kim et al. (2023) explored a potential mechanism underlying this relationship by focusing on autonomy, a well-established correlate of positive affect and well-being. Their experiment compared the effects of creative ideation versus non-creative ideation on feelings of autonomy. Results revealed that creative ideation significantly increased participants’ sense of autonomy relative to non-creative ideation (Kim et al., 2023). Together, these experimental studies provide compelling causal evidence for the beneficial impact of creativity on well-being.

Building on the aforementioned correlational and experimental evidence linking creativity to well-being, Holinger and Kaufman (2024) developed and validated the Self-Beliefs of Creativity and Well-Being (SBCW) scale. SBCW is embedded within the broader construct of creative self-belief, a concept that has been extensively investigated over several decades (Ginns et al., 2023; Karwowski and Kaufman, 2017; Karwowski and Lebuda, 2016; Karwowski et al., 2019). Creative self-beliefs refer to individuals’ perceptions of their own creativity and the extent to which they view themselves as creative (Karwowski and Barbot, 2016). However, Beghetto and Karwowski (2023) expanded this conceptualization, proposing that creative self-beliefs serve as a bridge linking creative potential to creative action. They identified three core dimensions: creative confidence, creative self-awareness, and creative self-image. Of these, creative self-image encompasses beliefs about both the perceived value of creativity and its potential risks. This perspective aligns with the growing recognition of a close association between creativity and well-being, particularly the notion that creativity may be positively associated with well-being, which has informed the development of the SBCW scale. This instrument conceptualizes well-being as two distinct yet complementary facets: short-term and long-term, and measures individuals’ perceptions of creativity’s potential benefits for each. Exploring well-being across different timeframes is informative, as long-term and short-term well-being emphasize distinct yet important life outcomes and entail different conceptions of the “good life.” Short-term well-being tends to define a good life as a satisfactory life, one marked by momentary pleasure and the fulfillment of immediate desires. In contrast, long-term well-being views a good life as a meaningful life, centered on purpose, personal growth, and enduring fulfillment (Liu et al., 2025). Specifically, short-term well-being, aligned with hedonic principles, emphasizes maximizing immediate positive affect through momentary rewards. Long-term well-being, rooted in eudaimonic traditions, centers on pursuing long-term goals congruent with one’s inherent potentials, core personal values, and meaning-making processes. Holinger and Kaufman (2024) further found that SBCW scores were positively associated with creative self-efficacy, defined as an individual’s belief in their capacity to generate novel, original, and contextually appropriate ideas or solutions (Beghetto, 2006). Extending this work cross-culturally, Liu et al. (2025) validated a Chinese version of the SBCW and expanded its nomological network. Their findings confirmed that SBCW was positively related to multiple dimensions of well-being, including life satisfaction, psychological richness, and meaning in life. Critically, they identified a mediating role of creative self-efficacy: it mediated the relationship between SBCW and three well-being outcomes, including life satisfaction, psychological richness, and presence of meaning, but not search for meaning (i.e., the active pursuit of life meaning). These results not only reinforce creativity’s relevance to well-being but also highlight the need to distinguish between distinct facets of well-being and their differential associations with SBCW. Notably, short-term SBCW directly predicted search for meaning, whereas long-term SBCW did not, providing empirical support for the discriminant validity of the SBCW’s short-term and long-term subscales. Despite these advancements, our understanding of SBCW remains limited, particularly from the perspective of self-reflective rumination.

### 1.3 The present study

As established earlier, prior literature demonstrates two key findings: first, self-reflective rumination is positively associated with creativity (Verhaeghen et al., 2005, 2014). Second, creativity exerts a facilitative effect on well-being, a relationship supported by both correlational and experimental evidence. Building on this foundation, Holinger and Kaufman (2024) recently developed and validated the Self-Beliefs of Creativity and Well-Being (SBCW), a novel construct that captures individuals’ beliefs about creativity’s potential benefits for their well-being (Holinger and Kaufman, 2024; Liu et al., 2025). However, a conceptual distinction exists between creativity as an ability/behavior and SBCW as a self-belief. To theoretically bridge this gap and justify examining a direct effect of SRR on SBCW, we draw upon the framework of Beghetto and Karwowski (2023). They posit that creative self-beliefs are not merely static traits but dynamic cognitive constructs that are shaped by experiences and, crucially, by reflective cognitive processes. Self-reflective rumination, characterized by deep, analytical thinking about one’s inner states and experiences, constitutes precisely such a process. Consequently, the primary goal of our study is to test this direct relationship between SRR and SBCW.

Beyond this direct association, further exploration of the mechanisms connecting self-reflective rumination to SBCW is warranted. Although Verhaeghen et al. (2005, 2014) confirmed a positive link between self-reflective rumination and creativity, the psychological pathways underlying this relationship remain unclear. We propose that creative self-efficacy, an individual’s belief in their capacity to generate novel, original, and contextually appropriate ideas (Tierney and Farmer, 2002), may serve as a critical mediating mechanism. This proposition is grounded in relevant empirical evidence: Fino and Sun (2022) identified creative self-efficacy as a mediator between personality traits (openness and conscientiousness) and mental well-being (Fino and Sun, 2022), while Dan et al. (2024) found that deliberate rumination predicts creativity via general self-efficacy (Dan et al., 2024). Notably, however, Dan et al. (2024) did not isolate the unique role of creative self-efficacy, a construct more specific to creative processes, in the rumination-creativity link. Addressing this gap, the second goal of the present study is to investigate whether creative self-efficacy mediates the association between self-reflective rumination and SBCW.

In summary, based on the aforementioned theoretical and empirical foundations, the present study addresses the following two research questions:

*RQ1*: What is the relationship between self-reflective rumination (SRR), creative self-efficacy (CS), and SBCW?

*RQ2*: Does creative self-efficacy play a mediating role in the association between self-reflective rumination and SBCW?

By addressing the aforementioned research questions, this inquiry offers valuable insights for clinical and counseling practitioners, underscoring the potential of self-reflective rumination as a pivotal catalyst fostering both creativity and well-being.

## 2 Methods

### 2.1 Design and context

The present study employed a cross-sectional design to investigate the interrelationships among self-reflective rumination, Self-Belief in Creativity and Well-Being (SBCW), and creative self-efficacy within the context of Chinese higher education.

### 2.2 Sampling and participants

Participants were recruited using convenience and snowball sampling methods. A total of 2,973 responses were initially collected; however, following an attention-check procedure, data from 1,993 participants were retained for analysis. Participants who failed to select the designated value (i.e., “4”) on the attention-check item (“Please choose 4 for this question”) were excluded. The final sample comprised 501 males, with a mean age of 20.96 years (SD = 2.87). The remaining sample did not differ significantly from the excluded participants in terms of age, *t* < 0.5, *p* > 0.10. However, a notable gender imbalance was observed: the retained sample consisted predominantly of female participants, whereas the excluded group had a higher proportion of males, χ^2^(1) = 62.0, *p* < 0.001. The majority of the remaining participants were undergraduates (93.13%), while the remainder consisted of master’s (5.52%) and doctoral students (1.35%). Most of the participants majored in Education (47.6%), Engineering (20.4%), Science (19.4%), Literature (7.2%), Art (2.9%), and others less than 1% participants majored in Management, Agriculture, and others. Based on the model specification with items in each measurement (see 2.3 below) and taking the path of primary interest (SRR → CS, *β* = 0.23, Cohen’s *f^2^* ≈ 0.056) as the benchmark, and setting the significance level at *α* = 0.05 and the desired statistical power at 0.80, the required sample size was estimated to be approximately 260 for detecting this effect; the sample size needed to evaluate the full model was approximately 300. The final analysis was based on 1993 valid participants, exceeding the minimum requirement.

### 2.3 Measurements

The Ruminative Response Scale (RRS) developed by Nolen-Hoeksema and Morrow (1991) was used to measure self-reflective rumination (Nolen-Hoeksema and Morrow, 1991). The RRS comprises three factors: symptom rumination, brooding, and reflective pondering. Given that self-reflective rumination (reflective pondering) is considered a potential positive driver of well-being, the present study focused primarily on this factor. Our research aim was to examine whether a potentially beneficial form of self-reflection could foster creativity-related beliefs and well-being. Accordingly, focusing on reflective pondering allowed us to test this hypothesis without confounding effects from the more negative ruminative styles. Self-reflective rumination was assessed using five items rated on a 7-point Likert scale (1 = totally disagree, 7 = totally agree), and this scale has been translated in Chinese and validated previously (Han, 2010). Reliability analysis confirmed good internal consistency (*α* = 0.90). Additionally, confirmatory factor analysis (CFA) demonstrated adequate construct validity for the scale, with fit indices as follows: CFI = 0.99, NFI = 0.99, IFI = 0.99, and RMSEA = 0.08. Creative self-efficacy was measured using the scale (5 items on a 7-point scale) from the study of Holinger and Kaufman (2024) which was based on the Beghetto’s (2006) study. This scale showed excellent reliability (*α* = 0.97) and validity in the present study (CFI = 1.00, NFI = 1.00, IFI = 1.00, RMSEA = 0.02). Its psychometric properties have also been validated in a previous study with a Chinese population (Liu et al., 2025). The Self-Beliefs in Creativity and Well-Being (SBCW) scale, developed by Holinger and Kaufman (2024), was used to assess self-beliefs in creativity and well-being. This scale has been validated in a Chinese context in a prior study without modification (Liu et al., 2025). In the present study, the SBCW scale exhibited satisfactory reliability: *α* = 0.96 for the SBCW-S subscale (7 items), *α* = 0.96 for the SBCW-L subscale (8 items), and *α* = 0.98 for the full SBCW scale. CFA results also confirmed adequate construct validity (CFI = 0.97, NFI = 0.97, IFI = 0.97, RMSEA = 0.09). The AVE for all constructs exceeded 0.5, indicating good convergent validity. Additionally, the correlations between constructs were lower than the square roots of their respective AVEs, which demonstrates good discriminant validity (Fornell and Larcker, 1981). The composite reliability (CR) values for each latent construct also exceeded 0.9, indicating a good reliability (Table 1).

**Table 1:** Descriptive statistics and correlations of all variables.

Variables	*M*	SD	Min	Max	Median	Skewness	Kurtosis	AVE	CR	1	2	3	4
1. SRR	4.04	1.08	1	7	4	−0.01	1.47	0.65	0.90	–			
2. CS	4.48	0.98	1	7	4	0.74	1.16	0.84	0.96	0.24^***^	–		
3. SBCW-L	4.63	0.97	1	7	4	0.87	0.37	0.77	0.97	0.21^***^	0.88^***^	–	
4. SBCW-S	4.69	1.01	1	7	4	0.81	0.11	0.80	0.96	0.23^***^	0.73^***^	0.80^***^	–

SRR, self-reflective rumination; CS, creative self-efficacy; SBCW-L, self-beliefs in creativity and well-being long term; SBCW-S, self-beliefs in creativity and well-being short term; AVE, average variance extracted, ^***^p < 0.001.

### 2.4 Procedure and ethics

After drafting the questionnaire, we distributed it via Wenjuanxing (wjx.cn), a widely used online survey platform in China, to college teachers from various universities across different regions of China. Before the data collection, the research protocol has been approved by the ethics committee in Teacher Education College, Suqian University.

### 2.5 Analytical strategies

Upon collecting the data, we first conducted descriptive statistical analyses of all variables and calculated their bivariate correlations. Second, we employed the structural equation modeling (SEM) function in JASP (Love et al., 2019) and jamovi (The Jamovi Project, 2024) to examine the mediating role of creative self-efficacy in the relationship between self-reflective rumination (SRR) and self-beliefs in creativity and well-being (SBCW). Given our large sample size (*N* = 1,993), which could provide high statistical power and may yield statistically significant effects even for small parameters, effect size estimates and confidence intervals were prioritized over *p*-values in interpreting results. Indirect effects were evaluated using bias-corrected and accelerated (BCa) bootstrap confidence intervals (5,000 resamples), a robust approach that focuses on parameter precision rather than null-hypothesis significance testing.

## 3 Results

Descriptive statistics for all study variables are summarized in Table 1. On average, participants reported moderately high levels of self-reflective rumination (SRR), creative self-efficacy (CS), and both short-term and long-term self-beliefs in creativity and well-being (SBCW-S and SBCW-L), with mean scores exceeding 4.0 on the 7-point Likert scales, indicating above-midpoint endorsement of these constructs.

Pearson correlation analyses (Table 1) showed that SRR was positively correlated with CS, SBCW-S, and SBCW-L. Importantly, the strength of the association between SRR and CS, as well as between SRR and SBCW, was notably weaker than the correlation observed between CS and SBCW, suggesting a closer conceptual linkage between creative self-efficacy and self-beliefs in creativity and well-being.

Following initial validation of each scale through unidimensional CFAs (see Section 2.3), we next tested a full measurement model comprising all latent constructs simultaneously. This comprehensive CFA would ensure the multi-construct measurement structure exhibit acceptable fit, supporting its use in the subsequent structural model. Model fit was assessed based on the following commonly recommended criteria: comparative fit index (CFI) and Tucker-Lewis index (TLI) values between 0.90 and 0.95 indicate acceptable fit, and values ≥ 0.95 indicate good fit; root mean square error of approximation (RMSEA) ≤ 0.08 indicates acceptable fit; and standardized root mean square residual (SRMR) < 0.10 indicates good fit (Schweizer, 2010). The results showed that the measurement model had an acceptable to good fit: CFI = 0.94, TLI = 0.93, RMSEA = 0.08, SRMR = 0.04. To assess common method bias (CMB), we implemented the unmeasured latent method construct (ULMC) approach (Podsakoff et al., 2024). A common method factor (CMF) was specified to load on all observed items, constrained to be uncorrelated with all theoretical constructs, and its variance fixed to 1 for model identification. Comparison of structural paths between the baseline model and the CMF model revealed negligible changes in all hypothesized relationships (maximum |Δ*β*| < 0.1), with all core paths retaining statistical significance.

**Figure 1:**
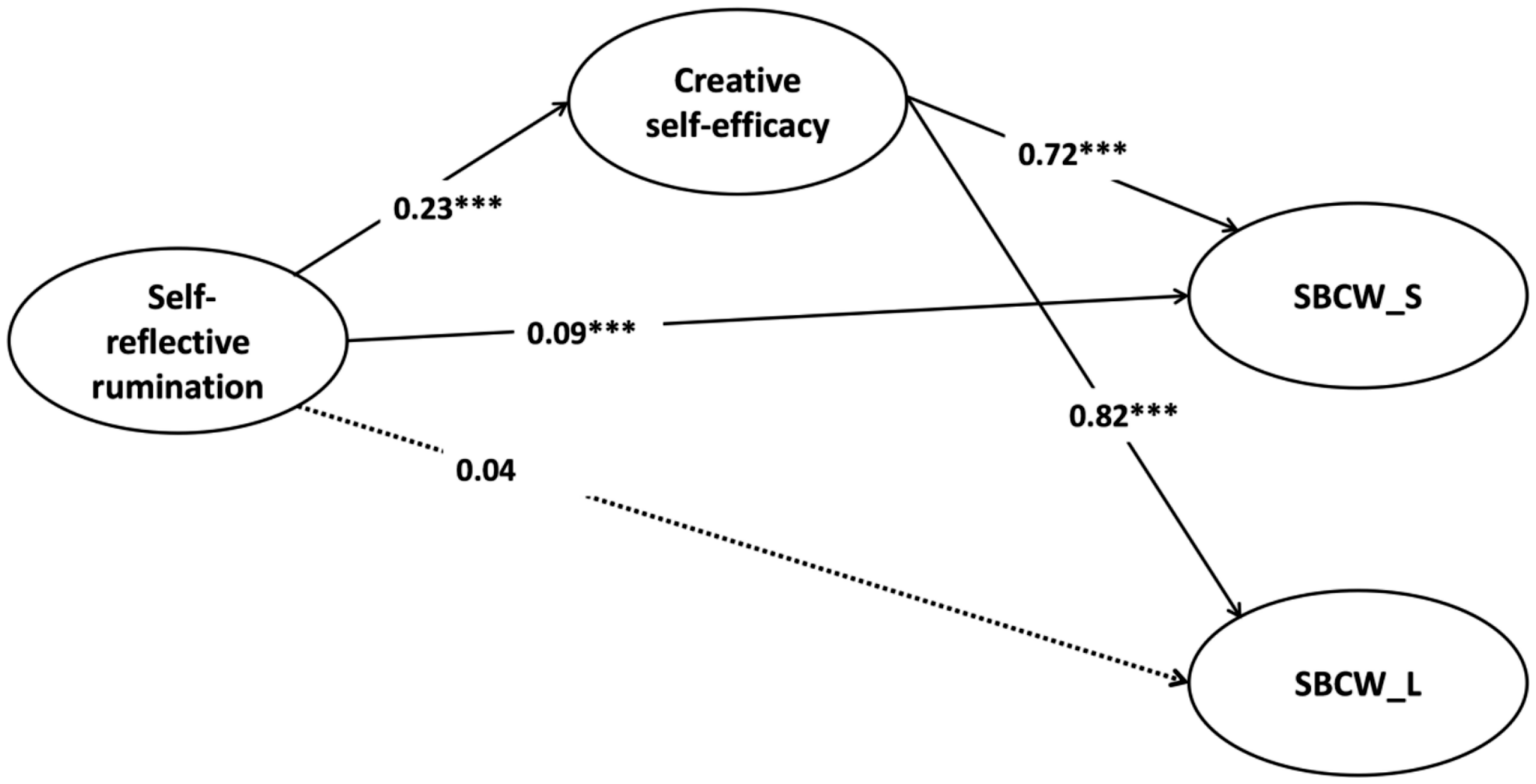
The mediation model. SBCW-L, self-beliefs in creativity and well-being long term; SBCW-S, self-beliefs in creativity and well-being short term, ^***^p < 0.001. Dashed lines indicate non-significant paths.

The structural model tested the mediation role of CS in the relationship between SRR and both SBCW-S and SBCW-L. Indirect effects (i.e., SRR → CS → SBCW-S and SRR → CEE → SBCW-L) were estimated using nonparametric bootstrapping with 5,000 resamples to compute standard errors and 95% bias-corrected and accelerated (BCa) confidence intervals (Gallucci and Jentschke, 2021; Jorgensen et al., 2019; Rosseel, 2012). Indirect effects were considered statistically significant if the BCa confidence interval did not include zero. Maximum likelihood (ML) estimation was used, with missing data handled via listwise deletion. In the structural model, SRR significantly predicted CS (*β* = 0.23, *z* = 6.39, *p* < 0.001). CS, in turn, significantly predicted both SBCW-S (*β* = 0.72, *z* = 24.31, *p* < 0.001) and SBCW-L (*β* = 0.82, *z* = 30.02, *p* < 0.001). Direct effects of SRR on SBCWS (*β* = 0.09, *z* = 3.38, *p* < 0.001) and SBCW-L (*β* = 0.04, *z* = 1.55, *p* = 0.122) were tested, with only the former reaching statistical significance (see Table 2 and Figure 1 for more details, we also conducted a model comparison to confirm the mediation effect in the Supplementary material).

**Table 2:** Direct effect of SRR on SBCW, and the indirect effect of CS.

Path	Direct effects	Indirect effects via CS
	[95% CI]	[95% CI]
SRR → SBCW-S	0.09^***^ [0.04, 0.13]	0.17^***^ [0.10, 0.20]
SRR → SBCW-L	0.04 [−0.00, 0.07]	0.19^***^ [0.12, 0.22]

SRR, self-reflective rumination; CS, creative self-efficacy; SBCW-L, self-beliefs in creativity and well-being long term; SBCW-S, self-beliefs in creativity and well-being short term, ^***^p < 0.001.

## 4 Discussion

The primary goal of the present study was to explore the role of self-reflective rumination in creativity and well-being, as well as the mediating role of creative self-efficacy in the relationships between self-reflective rumination and these two outcomes (creativity and well-being) that was measured by self-beliefs in creativity and well-being (SBCW). The results indicated that self-reflective rumination was positively associated with SBCW, encompassing both short-term and long-term dimensions. Furthermore, creative self-efficacy mediated the positive link between self-reflective rumination and SBCW. These findings not only underscore the significance of self-reflective rumination for creativity but also reveal that this facilitative effect of self-reflective rumination on creativity extends to short-term well-being. Creative self-efficacy, which is defined as an individual’s beliefs about their own creative abilities, emerges as a factor that connects both creativity and well-being, while also serving as a critical pathway connecting self-reflective rumination to SBCW.

Rumination was initially defined as a mode of responding to distress, characterized by repetitive, passive focus on the distress itself (Nolen-Hoeksema et al., 2008). However, deeper analysis of this construct revealed a positive dimension of rumination linked to creativity: its self-reflective, contemplative nature (Watkins, 2008). Self-reflective rumination involves purposefully engaging in cognitive problem-solving to alleviate distress, representing an adaptive form of rumination that supports recovery from depression (Treynor et al., 2003). Building on this adaptive understanding of rumination’s reflective component, researchers began exploring whether this type of rumination could facilitate creativity, an activity that inherently demands cognitive problem-solving and adaptive engagement with one’s internal states. Verhaeghen et al. (2005) initially proposed that the link between depression and creativity was explained by self-reflective rumination, which had direct effects on both. Verhaeghen et al. (2014) further refined the model by distinguishing rumination into brooding and reflective pondering. The results specifically demonstrated that reflective pondering indirectly predicted creative originality by positively linking to seriousness about creative activities (Verhaeghen et al., 2014).

The present study extends prior research by measuring self-beliefs in creativity and well-being (SBCW), a construct recently developed to assess individuals’ beliefs about creativity’s short-term and long-term benefits for well-being (Holinger and Kaufman, 2024). Our findings demonstrate that self-reflective rumination positively predicts both creativity and well-being, a connection increasingly documented in recent scholarship (Acar et al., 2021; Holinger and Kaufman, 2024; Liu et al., 2025). The SBCW was recently developed by Holinger and Kaufman (2024) and was intended to measure the belief of how creativity could be beneficial for well-being in long-term and short-term. Following the scale’s development, emerging research has explored SBCW’s contributions to multiple facets of well-being, including life satisfaction, meaning in life (King and Hicks, 2021), and psychological richness (Oishi and Westgate, 2022). Liu et al. (2025) found that short-term self-beliefs in creativity and well-being (SBCW) directly predict life satisfaction and meaning in life, but not psychological richness. In contrast, long-term SBCW directly predicts life satisfaction, psychological richness, and the presence of meaning, though not the search for meaning. These results not only highlight the distinction between short-term and long-term SBCW but also reveal that beliefs about creativity’s role in well-being exert multifaceted influences on well-being outcomes. Notably, Liu et al. (2025) did not explore the antecedents of SBCW. Building on prior literature, we hypothesized that self-reflective rumination might function as an antecedent of SBCW. The present study confirms this speculation: self-reflective rumination predicts short-term SBCW. These findings extend prior research on the link between self-reflective rumination and creativity by demonstrating that its positive correlation with creative behavior also contributes to well-being, thereby broadening the theoretical scope of rumination’s adaptive functions. This expanded view aligns with the Creative-Being Model (Beresford et al., 2025), which posits that self-reflective rumination “enables positive transformative processes through the adaptive nature of reflection” and is thus “key to promoting creative behavior.” While this proposition was originally advanced through conceptual analysis, the present study provides empirical support for it. However, the direct effect of self-reflective rumination (SRR) on long-term SBCW-L was not statistically significant, suggesting that SRR may influence well-being primarily in the short term, such as through immediate affective states or life satisfaction, rather than through enduring perceptions of meaning in life. This finding also highlights a meaningful distinction between SBCW-Land SBCW-S, consistent with recent evidence reported by Liu et al. (2025). Despite the absence of a significant direct effect on SBCW-L, a robust indirect effect emerged via creative self-efficacy (CS), which would be discussed below.

Beyond establishing a direct positive association between self-reflective rumination and SBCW, we also found that creative self-efficacy partially mediates this relationship. This builds upon recent work by Liu et al. (2025), who demonstrated that creative self-efficacy mediates the link between SBCW and multiple facets of well-being. Our results extend this mediation model by positioning creative self-efficacy as a crucial mechanism connecting self-reflective rumination to creativity-oriented well-being outcomes. This suggests that creative self-efficacy, defined as one’s belief in their capacity to generate novel and useful ideas, plays a pivotal role across contexts, whether creativity functions as an independent or dependent variable. This is consistent with the process-oriented perspective on creative self-efficacy (Puente-Díaz, 2016), which emphasizes its dynamic role in facilitating creative engagement. Moreover, the mediating role of creative self-efficacy deepens our understanding of how self-reflective rumination fosters creativity. Unlike brooding, which fixates on problems without seeking solutions, self-reflective rumination is oriented toward problem-solving and self-regulation, enhancing self-knowledge and psychological adaptiveness (Takano and Tanno, 2009). This constructive introspection may bolster individuals’ confidence in their creative capabilities, thereby increasing creative self-efficacy and, in turn, promoting creativity across diverse settings. The findings indicate that self-reflective rumination is not only associated with creativity but can also be positively related to individuals’ self-beliefs in creativity and well-being (SBCW) by a positive association with creative self-efficacy. This insight has implications for integrating adaptive reflection into mental health and creativity cultivation practices. For example, developing structured self-reflection rumination, such as through reflective portfolios, could be integrated into curricula to help students recognize their creative capacities, thereby strengthening their belief that creativity is not only attainable but also conducive to personal well-being.

Despite its contributions, the present study has several limitations that warrant further investigation. First, our sample consisted exclusively of participants from China. Given the cultural emphasis on self-reflection in Chinese society, rooted in Confucian teachings such as “I reflect on myself three times each day” (Wu ri san xing wu shen), a maxim widely taught across generations, it is possible that our findings may not generalize to individuals from other cultural contexts. In cultures where introspection is less normative or valued differently, the adaptive role of self-reflective rumination in fostering creativity and well-being might differ. Future research should therefore examine whether the positive link between self-reflective rumination and subjective well-being through creative work (SBCW) holds across diverse cultural backgrounds. Second, the study focused solely on self-reflective rumination, while other psychological antecedents of SBCW remain underexplored. For instance, Grant et al. (2002) distinguished self-reflection from insight: the former refers to active thoughts about one’s thoughts or feelings (regardless of conscious awareness), whereas the latter denotes a deeper internal awareness and understanding of one’s mental and emotional states (Grant et al., 2002). It is plausible that insight may moderate or mediate the relationship between self-reflective rumination and creativity. Future studies could investigate this possibility to clarify the distinct and interactive roles of reflective processes in creative development. Third, although our study focused on self-reflective rumination, other forms of rumination, such as brooding and symptom rumination, warrant further investigation to elucidate the independent and interactive effects of different ruminative styles on well-being and creativity. This represents a promising direction for future research. Fourth, although attention check questions were employed to exclude disengaged participants and enhance data quality, significant demographic differences (e.g., gender distribution) were observed between retained and excluded subsamples. Consequently, caution should be exercised when generalizing these findings to populations with notably different gender compositions, especially those with a higher proportion of males. To strengthen the external validity of these results and address the limitations inherent in cross-sectional designs, such as the inability to infer temporal or causal relationships, we recommend that future research replicate these analyses using more demographically diverse and representative samples. Additionally, employing a longitudinal design could help to overcome the shortcomings of cross-sectional studies by delineating the temporal and causal relationships between creativity, well-being, and various personal factors.

## Data Availability

The raw data supporting the conclusions of this article will be made available by the authors, without undue reservation.
