# Supplementary material for: “Looking inward,” creativity, and well-being: an SEM analysis of self-reflective rumination and creative self-efficacy as pathways linking self-beliefs in creativity and well-being in college students
Source: Front Psychol. 2026 May 28;17:1776026. doi: 10.3389/fpsyg.2026.1776026 (PMC13254061; doi:10.3389/fpsyg.2026.1776026)
Supplement: Supplementary file 1 [file Table_1.docx]

**Supplementary materials**

**Model comparison for mediation effect**

To assess the necessity of including creative self-efficacy (CS) as a mediator, we conducted a nested model comparison. Model 1 included all hypothesized paths (SSR → CS, CS → SBCW-S/SBCW-L), while Model 2 constrained the indirect paths to zero (i.e., no mediation). The chi-square difference test indicated that Model 1 provided a significantly better fit to the data than Model 2, Δχ²(2) = 16.538, p < .001. Additionally, Model 1 demonstrated superior fit indices: SRMR = 0.035 vs 0.038, RMSEA = 0.053 vs. 0.054, and SRMR = 0.035 vs. 0.038 (see Table. S.1). These results support the inclusion of CS as a significant mediator in the relationship between self-reflective rumination and self-beliefs in creativity and well-being (SBCW).

Table. S.1 Fit indices for nested model comparison

| Model | Description | χ² | df | Δχ²(Δdf) | p (Δχ²) |
| --- | --- | --- | --- | --- | --- |
| Model 1 | Full mediation model  (RRSR → CS → SBCW-S/L) | 371.02 | 267 |  |  |
| Model 2 | Direct-effects-only model  (Mediation paths constrained to zero) | 433.72 | 269 | 16.54(2) | < 0.001 |

Table. S.2 Correlation matrix and descriptive statistics (M ± SD) for females and males (upper side)

|  | Female | Male | 1 | 2 | 3 | 4 |
| --- | --- | --- | --- | --- | --- | --- |
| 1. SRR | 4.02 ± 1.10 | 4.08 ± 1.02 | ___ | 0.26^***^ | 0.29^***^ | 0.28^***^ |
| 1. CS | 4.40 ± 0.98 | 4.40 ± 0.98 | 0.21^***^ | ___ | 0.64^***^ | 0.69^***^ |
| 1. SBCW-S | 4.55 ± 1.00 | 4.55 ± 1.00 | 0.19^***^ | 0.61^***^ | ___ | 0.74^***^ |
| 1. SBCW-L | 4.49 ± 0.95 | 4.49 ± 0.95 | 0.19^***^ | 0.67^***^ | 0.77^***^ | ___ |

Note. ^***^ *p* < 0.001

**Model comparison of adding gender and age**

To assess robustness, we controlled for gender and age as demographic covariates in an alternative model. Model comparison revealed no significant deterioration in fit, and all hypothesized structural paths remained substantively unchanged (|Δβ| < .02). These results confirm that demographic factors did not alter theoretical conclusions. Full comparison statistics are reported in Supplementary Material (Table. S.3).

Table. S.3 Fit indices for nested model comparison

| Model | Description | χ² | df | Δχ²(Δdf) | p (Δχ²) |
| --- | --- | --- | --- | --- | --- |
| Model 1 | Full model with gender and age | 4102.93 | 288 |  |  |
| Model 2 | Our hypothesized model | 4157.39 | 330 | 54.46(42) | 0.09 |
